# (2-Oxido-1-naphthaldehyde benzoyl­hydrazonato-κ^3^ *N*,*N*′,*O*)pyridine­copper(II)

**DOI:** 10.1107/S1600536811011081

**Published:** 2011-03-31

**Authors:** Li-Fei Zou, Xiu-Yun Yang, Ying Gao, Hai-Bo Yao, Yun-Hui Li

**Affiliations:** a School of Chemistry and Environmental Engineering, Changchun University of Science and Technology, Changchun, 130022, People’s Republic of China

## Abstract

In the mononuclear title compound, [Cu^II^(C_18_H_12_N_2_O_2_)(C_5_H_5_N)], the Cu^II^ ion is coordinated by two O atoms and one N atom from the dianionic tridentate *L* ^2−^ ligand (H_2_ *L* is 2-hy­droxy-1-naphthaldehyde benzoyl­hydrazide) and one N atom from a pyridine mol­ecule in a CuN_2_O_2_ distorted square-planar coordination environment.

## Related literature

For the preparation of the Schiff base, see: Qiao *et al.* (2010 ▶). For chemically related applications arising from Schiff base compounds, see: Ando *et al.* (2004 ▶); Anford *et al.* (1998 ▶); Guo *et al.* (2010 ▶). For related structures, see: Ali *et al.* (2004 ▶); Sun *et al.* (2011 ▶); Xu *et al.* (2006 ▶); Yu *et al.* (2010 ▶).
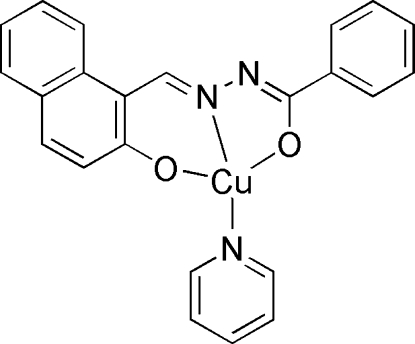


## Experimental

### 

#### Crystal data

[Cu(C_18_H_12_N_2_O_2_)(C_5_H_5_N)]
*M* *_r_* = 430.94
Monoclinic, 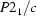

*a* = 11.6196 (6) Å
*b* = 8.4254 (4) Å
*c* = 19.6194 (10) Å
β = 106.247 (1)°
*V* = 1844.03 (16) Å^3^
*Z* = 4
Mo *K*α radiation
μ = 1.21 mm^−1^
*T* = 185 K
0.14 × 0.12 × 0.10 mm

#### Data collection

Bruker APEXII CCD area detector diffractometer
Absorption correction: multi-scan (*SADABS*; Bruker, 2004 ▶) *T*_min_ = 0.849, *T*_max_ = 0.889
8559 measured reflections
3619 independent reflections
2860 reflections with *I* > 2σ(*I*)
*R*_int_ = 0.031

#### Refinement

*R*[*F* ^2^ > 2σ(*F* ^2^)] = 0.036
*wR*(*F* ^2^) = 0.086
*S* = 1.03
3619 reflections
262 parameters
H-atom parameters constrained
Δρ_max_ = 0.46 e Å^−3^
Δρ_min_ = −0.29 e Å^−3^

### 

Data collection: *APEX2* (Bruker, 2004 ▶); cell refinement: *SAINT* (Bruker, 2004 ▶); data reduction: *SAINT*; program(s) used to solve structure: *SHELXS97* (Sheldrick, 2008 ▶); program(s) used to refine structure: *SHELXL97* (Sheldrick, 2008 ▶); molecular graphics: *DIAMOND* (Brandenburg, 1999 ▶); software used to prepare material for publication: *SHELXL97* and *publCIF* (Westrip, 2010 ▶).

## Supplementary Material

Crystal structure: contains datablocks I, global. DOI: 10.1107/S1600536811011081/zq2095sup1.cif

Structure factors: contains datablocks I. DOI: 10.1107/S1600536811011081/zq2095Isup2.hkl

Additional supplementary materials:  crystallographic information; 3D view; checkCIF report

## Figures and Tables

**Table:** 

Cu1—O1	1.8853 (17)
Cu1—N1	1.902 (2)
Cu1—O2	1.9243 (17)
Cu1—N3	2.001 (2)

**Table:** 

O1—Cu1—N1	93.17 (8)
O1—Cu1—O2	172.59 (7)
N1—Cu1—O2	81.30 (8)
O1—Cu1—N3	93.65 (8)
N1—Cu1—N3	171.07 (8)
O2—Cu1—N3	92.37 (8)

**Fig. 1.**
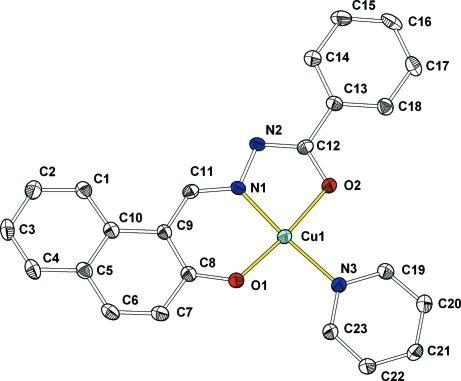
A view of the title compound, showing the atomic numbering scheme. Displacement ellipsoids are drawn at the 40% probability level.

## supplementary crystallographic information

### Comment

Interest in the chemistry of Schiff base arises from their ability to bind to metal ions (Yu *et al.*, 2010) as well as their antitumor activities and magnetochemistry (Ando *et al.*, 2004; Guo *et al.*, 2010). In fact, with some acylhydrazone ligands, their metal compounds are endowed with significantly improved industrial processes (Anford *et al.*, 1998). We selected the 2-hydroxy-1-naphthaldehyde benzoylhydrazide (H_2_*L*) Schiff base ligand to construct coordination polymers with defined geometry, due to its combination of nitrogen and oxygen donor atoms. We report here the preparation and crystal structure of the Schiff base Cu^II^ title compound.

The present compound, [Cu^II^(C_18_H_12_N_2_O_2_)(C_5_H_5_N)], together with the atom-numbering scheme, is illustrated in Fig. 1. Selected bond lengths and angles are given in Table 1. The Cu^II^ ion is coordinated by two O atoms and one N atom from the dianionic tridentate ligand *L*^2-^ ligand (H_2_*L* is 2-hydroxy-1-naphthaldehyde benzoylhydrazide), and one N atom from a pyridine molecule. The Cu^II^ ion adopts a CuN_2_O_2_ distorted square-planar coordination environment. The Cu—O and Cu—N bond distances are similar to the corresponding bond distances observed in related compounds (Ali *et al.*, 2004; Xu *et al.*, 2006; Sun *et al.*, 2011). There is no significant deviation of the metal centre from the N_2_O_2_ equatorial plane. The maximum displacements from the least-squares plane through N1, N3, O1, and O2 (rms deviation = 0.0895 Å) are 0.096 (1) and -0.094 (1) Å for atoms N1 and O2; Cu1 is -0.013 (1) Å below the mean plane. The coordinated pyridine molecule is almost coplanar with the previous N_2_O_2_ plane, the dihedral angle between the mean planes is 7.2 (1)°.

### Experimental

The 2-hydroxy-1-naphthaldehyde benzoylhydrazide ligand (H_2_*L*) was prepared in a similar manner to the reported procedures (Qiao *et al.*, 2010). The title compound was synthesized by adding pyridine (0.2 mL) to a solution of H_2_*L* (0.1 mmol) and Cu(OAc)_2_ (0.1 mmol) in methanol/dichloromethane (1:1, 20 mL), and the resulting mixture was stirred for about 6 h to afford a green solution. A week later, brown crystals of the title compound were isolated from the solution.

### Refinement

All H atoms were placed in calculated positions and refined using a riding model [C–H = 0.95 Å and *U*_iso_(H) = 1.5*U*_eq_(C)].

### Crystal data

**Table:**

[Cu(C_18_H_12_N_2_O_2_)(C_5_H_5_N)]	*F*(000) = 884
*M**_r_* = 430.94	*D*_x_ = 1.552 Mg m^−^^3^
Monoclinic, *P*2_1_/*c*	Mo *K*α radiation, λ = 0.71073 Å
Hall symbol: -P 2ybc	Cell parameters from 9033 reflections
*a* = 11.6196 (6) Å	θ = 4.8–51.9°
*b* = 8.4254 (4) Å	µ = 1.21 mm^−^^1^
*c* = 19.6194 (10) Å	*T* = 185 K
β = 106.247 (1)°	Block, brown
*V* = 1844.03 (16) Å^3^	0.14 × 0.12 × 0.10 mm
*Z* = 4	

### Data collection

**Table:**

Bruker APEXII CCD area detector diffractometer	3619 independent reflections
Radiation source: fine-focus sealed tube	2860 reflections with *I* > 2σ(*I*)
graphite	*R*_int_ = 0.031
phi and ω scans	θ_max_ = 26.0°, θ_min_ = 1.8°
Absorption correction: multi-scan (*SADABS*; Bruker, 2004)	*h* = −14→14
*T*_min_ = 0.849, *T*_max_ = 0.889	*k* = −6→10
8559 measured reflections	*l* = −18→24

### Refinement

**Table:**

Refinement on *F*^2^	Primary atom site location: structure-invariant direct methods
Least-squares matrix: full	Secondary atom site location: difference Fourier map
*R*[*F*^2^ > 2σ(*F*^2^)] = 0.036	Hydrogen site location: inferred from neighbouring sites
*wR*(*F*^2^) = 0.086	H-atom parameters constrained
*S* = 1.03	*w* = 1/[σ^2^(*F*_o_^2^) + (0.0353*P*)^2^ + 1.0945*P*] where *P* = (*F*_o_^2^ + 2*F*_c_^2^)/3
3619 reflections	(Δ/σ)_max_ = 0.001
262 parameters	Δρ_max_ = 0.46 e Å^−^^3^
0 restraints	Δρ_min_ = −0.29 e Å^−^^3^

### Special details

**Table:**

Geometry. All esds (except the esd in the dihedral angle between two l.s. planes) are estimated using the full covariance matrix. The cell esds are taken into account individually in the estimation of esds in distances, angles and torsion angles; correlations between esds in cell parameters are only used when they are defined by crystal symmetry. An approximate (isotropic) treatment of cell esds is used for estimating esds involving l.s. planes.
Refinement. Refinement of *F*^2^ against ALL reflections. The weighted R-factor wR and goodness of fit *S* are based on *F*^2^, conventional *R*-factors *R* are based on *F*, with *F* set to zero for negative *F*^2^. The threshold expression of *F*^2^ > 2sigma(*F*^2^) is used only for calculating R-factors(gt) etc. and is not relevant to the choice of reflections for refinement. *R*-factors based on *F*^2^ are statistically about twice as large as those based on *F*, and *R*- factors based on ALL data will be even larger.

### Fractional atomic coordinates and isotropic or equivalent isotropic displacement parameters (Å^2^)

**Table:**

	*x*	*y*	*z*	*U*_iso_*/*U*_eq_	
Cu1	0.86976 (3)	0.13588 (4)	0.539632 (16)	0.02191 (11)	
N1	0.89789 (18)	0.1188 (2)	0.44895 (11)	0.0200 (5)	
N2	0.99966 (18)	0.1976 (3)	0.44156 (11)	0.0216 (5)	
N3	0.86766 (19)	0.1483 (2)	0.64115 (11)	0.0226 (5)	
O1	0.72604 (15)	0.0186 (2)	0.51138 (9)	0.0247 (4)	
O2	1.00963 (15)	0.2688 (2)	0.55668 (9)	0.0238 (4)	
C1	0.7084 (2)	−0.1291 (3)	0.26471 (14)	0.0265 (6)	
H1	0.7807	−0.0755	0.2655	0.032*	
C2	0.6465 (3)	−0.2069 (3)	0.20399 (14)	0.0300 (6)	
H2	0.6762	−0.2046	0.1635	0.036*	
C3	0.5411 (3)	−0.2891 (3)	0.20059 (15)	0.0306 (6)	
H3	0.4997	−0.3440	0.1586	0.037*	
C4	0.4982 (3)	−0.2894 (3)	0.25876 (15)	0.0313 (7)	
H4	0.4259	−0.3444	0.2567	0.038*	
C5	0.5588 (2)	−0.2100 (3)	0.32154 (14)	0.0261 (6)	
C6	0.5135 (2)	−0.2110 (3)	0.38178 (15)	0.0295 (6)	
H6	0.4416	−0.2672	0.3794	0.035*	
C7	0.5702 (2)	−0.1339 (3)	0.44258 (14)	0.0267 (6)	
H7	0.5366	−0.1366	0.4815	0.032*	
C8	0.6791 (2)	−0.0489 (3)	0.44957 (13)	0.0221 (6)	
C9	0.7276 (2)	−0.0447 (3)	0.39116 (13)	0.0205 (5)	
C10	0.6671 (2)	−0.1266 (3)	0.32608 (13)	0.0225 (6)	
C11	0.8363 (2)	0.0379 (3)	0.39455 (13)	0.0216 (6)	
H11	0.8658	0.0333	0.3541	0.026*	
C12	1.0481 (2)	0.2750 (3)	0.50057 (13)	0.0213 (6)	
C13	1.1522 (2)	0.3790 (3)	0.50397 (14)	0.0219 (6)	
C14	1.1922 (3)	0.4090 (3)	0.44463 (15)	0.0329 (7)	
H14	1.1537	0.3597	0.4006	0.039*	
C15	1.2877 (3)	0.5103 (4)	0.44947 (16)	0.0385 (7)	
H15	1.3142	0.5301	0.4086	0.046*	
C16	1.3449 (3)	0.5828 (3)	0.51292 (17)	0.0361 (7)	
H16	1.4105	0.6522	0.5158	0.043*	
C17	1.3063 (2)	0.5537 (3)	0.57197 (16)	0.0328 (7)	
H17	1.3455	0.6032	0.6158	0.039*	
C18	1.2107 (2)	0.4527 (3)	0.56769 (14)	0.0262 (6)	
H18	1.1847	0.4334	0.6087	0.031*	
C19	0.9510 (2)	0.2352 (3)	0.68806 (15)	0.0323 (7)	
H19	1.0071	0.2946	0.6714	0.039*	
C20	0.9577 (3)	0.2408 (4)	0.75936 (15)	0.0350 (7)	
H20	1.0176	0.3027	0.7911	0.042*	
C21	0.8762 (3)	0.1554 (3)	0.78380 (14)	0.0313 (7)	
H21	0.8795	0.1564	0.8327	0.038*	
C22	0.7900 (3)	0.0686 (4)	0.73614 (15)	0.0332 (7)	
H22	0.7319	0.0101	0.7515	0.040*	
C23	0.7889 (2)	0.0676 (3)	0.66537 (14)	0.0275 (6)	
H23	0.7294	0.0067	0.6328	0.033*	

### Atomic displacement parameters (Å^2^)

**Table:**

	*U*^11^	*U*^22^	*U*^33^	*U*^12^	*U*^13^	*U*^23^
Cu1	0.02087 (18)	0.02664 (19)	0.01893 (17)	−0.00342 (14)	0.00672 (13)	−0.00051 (14)
N1	0.0180 (11)	0.0199 (11)	0.0215 (11)	−0.0023 (9)	0.0045 (9)	0.0025 (9)
N2	0.0180 (11)	0.0242 (11)	0.0227 (11)	−0.0027 (9)	0.0062 (9)	0.0032 (9)
N3	0.0222 (11)	0.0258 (12)	0.0204 (11)	−0.0017 (10)	0.0069 (9)	−0.0012 (9)
O1	0.0248 (10)	0.0274 (10)	0.0233 (10)	−0.0047 (8)	0.0092 (8)	−0.0023 (8)
O2	0.0240 (10)	0.0289 (10)	0.0201 (9)	−0.0047 (8)	0.0085 (8)	−0.0025 (8)
C1	0.0250 (14)	0.0242 (14)	0.0281 (14)	0.0001 (12)	0.0037 (12)	0.0004 (12)
C2	0.0352 (16)	0.0268 (15)	0.0262 (15)	0.0026 (13)	0.0053 (13)	−0.0006 (12)
C3	0.0333 (16)	0.0248 (15)	0.0277 (15)	0.0017 (13)	−0.0016 (13)	−0.0075 (12)
C4	0.0269 (15)	0.0256 (15)	0.0353 (16)	−0.0040 (12)	−0.0011 (13)	−0.0040 (13)
C5	0.0266 (15)	0.0196 (14)	0.0296 (15)	−0.0009 (11)	0.0038 (12)	0.0018 (12)
C6	0.0249 (15)	0.0262 (15)	0.0367 (17)	−0.0071 (12)	0.0073 (13)	0.0012 (13)
C7	0.0243 (14)	0.0267 (15)	0.0298 (15)	−0.0027 (12)	0.0086 (12)	0.0036 (12)
C8	0.0223 (13)	0.0189 (13)	0.0235 (14)	0.0012 (11)	0.0039 (11)	0.0027 (11)
C9	0.0202 (13)	0.0175 (13)	0.0217 (13)	0.0002 (10)	0.0025 (11)	0.0018 (10)
C10	0.0219 (13)	0.0178 (13)	0.0249 (14)	0.0031 (11)	0.0018 (11)	0.0029 (11)
C11	0.0248 (14)	0.0208 (13)	0.0201 (13)	0.0033 (11)	0.0076 (11)	0.0037 (11)
C12	0.0221 (14)	0.0214 (14)	0.0208 (13)	0.0040 (11)	0.0067 (11)	0.0038 (11)
C13	0.0199 (13)	0.0195 (13)	0.0261 (14)	0.0014 (11)	0.0061 (11)	0.0034 (11)
C14	0.0372 (17)	0.0342 (16)	0.0293 (16)	−0.0103 (13)	0.0127 (13)	−0.0024 (13)
C15	0.0434 (19)	0.0386 (18)	0.0400 (18)	−0.0107 (15)	0.0222 (15)	0.0020 (14)
C16	0.0311 (16)	0.0264 (15)	0.054 (2)	−0.0115 (13)	0.0172 (15)	−0.0010 (14)
C17	0.0294 (16)	0.0286 (16)	0.0384 (17)	−0.0077 (13)	0.0063 (13)	−0.0087 (13)
C18	0.0271 (15)	0.0243 (14)	0.0284 (15)	−0.0013 (12)	0.0096 (12)	−0.0040 (12)
C19	0.0306 (16)	0.0393 (17)	0.0291 (15)	−0.0132 (14)	0.0119 (13)	−0.0048 (13)
C20	0.0385 (17)	0.0398 (18)	0.0264 (15)	−0.0112 (14)	0.0087 (13)	−0.0066 (13)
C21	0.0429 (17)	0.0316 (16)	0.0214 (14)	−0.0048 (13)	0.0120 (13)	−0.0026 (12)
C22	0.0374 (17)	0.0374 (17)	0.0287 (15)	−0.0117 (14)	0.0156 (13)	−0.0043 (13)
C23	0.0268 (15)	0.0327 (15)	0.0235 (14)	−0.0072 (12)	0.0075 (12)	−0.0031 (12)

### Geometric parameters (Å, °)

**Table:**

Cu1—O1	1.8853 (17)	C8—C9	1.412 (3)
Cu1—N1	1.902 (2)	C9—C11	1.428 (3)
Cu1—O2	1.9243 (17)	C9—C10	1.449 (3)
Cu1—N3	2.001 (2)	C11—H11	0.9500
N1—C11	1.300 (3)	C12—C13	1.480 (3)
N1—N2	1.398 (3)	C13—C18	1.391 (4)
N2—C12	1.311 (3)	C13—C14	1.392 (4)
N3—C23	1.331 (3)	C14—C15	1.381 (4)
N3—C19	1.349 (3)	C14—H14	0.9500
O1—C8	1.312 (3)	C15—C16	1.379 (4)
O2—C12	1.300 (3)	C15—H15	0.9500
C1—C2	1.374 (4)	C16—C17	1.376 (4)
C1—C10	1.415 (4)	C16—H16	0.9500
C1—H1	0.9500	C17—C18	1.384 (4)
C2—C3	1.392 (4)	C17—H17	0.9500
C2—H2	0.9500	C18—H18	0.9500
C3—C4	1.367 (4)	C19—C20	1.380 (4)
C3—H3	0.9500	C19—H19	0.9500
C4—C5	1.405 (4)	C20—C21	1.378 (4)
C4—H4	0.9500	C20—H20	0.9500
C5—C6	1.422 (4)	C21—C22	1.374 (4)
C5—C10	1.423 (4)	C21—H21	0.9500
C6—C7	1.356 (4)	C22—C23	1.385 (4)
C6—H6	0.9500	C22—H22	0.9500
C7—C8	1.427 (4)	C23—H23	0.9500
C7—H7	0.9500		
			
O1—Cu1—N1	93.17 (8)	C1—C10—C5	116.4 (2)
O1—Cu1—O2	172.59 (7)	C1—C10—C9	124.1 (2)
N1—Cu1—O2	81.30 (8)	C5—C10—C9	119.6 (2)
O1—Cu1—N3	93.65 (8)	N1—C11—C9	124.8 (2)
N1—Cu1—N3	171.07 (8)	N1—C11—H11	117.6
O2—Cu1—N3	92.37 (8)	C9—C11—H11	117.6
C11—N1—N2	116.8 (2)	O2—C12—N2	124.3 (2)
C11—N1—Cu1	127.48 (17)	O2—C12—C13	117.2 (2)
N2—N1—Cu1	115.64 (15)	N2—C12—C13	118.6 (2)
C12—N2—N1	108.15 (19)	C18—C13—C14	118.5 (2)
C23—N3—C19	117.9 (2)	C18—C13—C12	119.5 (2)
C23—N3—Cu1	121.99 (18)	C14—C13—C12	122.0 (2)
C19—N3—Cu1	120.08 (17)	C15—C14—C13	120.3 (3)
C8—O1—Cu1	127.16 (16)	C15—C14—H14	119.9
C12—O2—Cu1	110.51 (16)	C13—C14—H14	119.9
C2—C1—C10	121.7 (3)	C16—C15—C14	120.7 (3)
C2—C1—H1	119.2	C16—C15—H15	119.6
C10—C1—H1	119.2	C14—C15—H15	119.6
C1—C2—C3	121.3 (3)	C17—C16—C15	119.5 (3)
C1—C2—H2	119.4	C17—C16—H16	120.2
C3—C2—H2	119.4	C15—C16—H16	120.2
C4—C3—C2	118.8 (3)	C16—C17—C18	120.2 (3)
C4—C3—H3	120.6	C16—C17—H17	119.9
C2—C3—H3	120.6	C18—C17—H17	119.9
C3—C4—C5	121.4 (3)	C17—C18—C13	120.7 (3)
C3—C4—H4	119.3	C17—C18—H18	119.6
C5—C4—H4	119.3	C13—C18—H18	119.6
C4—C5—C6	121.0 (3)	N3—C19—C20	122.5 (3)
C4—C5—C10	120.5 (3)	N3—C19—H19	118.8
C6—C5—C10	118.5 (2)	C20—C19—H19	118.8
C7—C6—C5	121.8 (2)	C21—C20—C19	119.0 (3)
C7—C6—H6	119.1	C21—C20—H20	120.5
C5—C6—H6	119.1	C19—C20—H20	120.5
C6—C7—C8	121.7 (2)	C22—C21—C20	118.8 (3)
C6—C7—H7	119.2	C22—C21—H21	120.6
C8—C7—H7	119.2	C20—C21—H21	120.6
O1—C8—C9	125.6 (2)	C21—C22—C23	119.1 (3)
O1—C8—C7	115.8 (2)	C21—C22—H22	120.4
C9—C8—C7	118.6 (2)	C23—C22—H22	120.4
C8—C9—C11	121.6 (2)	N3—C23—C22	122.7 (3)
C8—C9—C10	119.9 (2)	N3—C23—H23	118.6
C11—C9—C10	118.6 (2)	C22—C23—H23	118.6
